# Clinicopathological Profile of Renal Cancer in a Caribbean Hospital: Analysis of a Surgical Case Series

**DOI:** 10.7759/cureus.17482

**Published:** 2021-08-27

**Authors:** Dinelle Sirjuesingh, Raye-Ann Sandy, Satyendra A Persaud

**Affiliations:** 1 Urology, San Fernando General Hospital, San Fernando, TTO; 2 Surgery, Scarborough General Hospital, Tobago, TTO; 3 Division of Clinical Surgical Sciences, University of the West Indies, St Augustine, TTO

**Keywords:** transitional cell carcinoma, oncology, caribbean, renal neoplasm, renal pathology

## Abstract

Objective

To document the demographic and pathological profile of renal cancer at San Fernando General Hospital (SFGH), Trinidad and Tobago over a five-year period (2015-2019).

Methods

This is a retrospective study that was conducted on all patients who had a histological diagnosis of renal cancer with surgical treatment from 2015-2019 at SFGH. Data were collected on patient demographics, clinical presentation, and pathological characteristics such as cancer size, location, and grade. Data were tabulated on Microsoft Excel and results were summarized using descriptive statistics.

Results

Over a 5-year period, there were 42 patients diagnosed with kidney cancer who had surgical intervention. The age ranged from 18 to 81 years with a mean age of 61 years and 67% of patients were over the age of 60. Males consisted of 57% of all patients. Most patients presented with pain, hematuria, or both. The majority (93%) of the patients had radical surgical treatment with equal distribution of right and left tumors. Clear cell carcinoma is the most common renal cell carcinoma (RCC), accounting for 80.5% followed by papillary with 16.7%. The majority of renal cell tumors were Fuhrman grade 2 with negative surgical margins and no lymphovascular invasion. The average maximum tumor dimension was 4.2 cm.

Conclusions

This study shows that in our hospital renal cancer affects primarily older patients, mostly men with the common presentation of pain and hematuria. The tumors are commonly clear cell RCC, grade 2 with negative margins, no lymphovascular invasion, and an average maximum dimension of 4.2 cm.

## Introduction

Kidney cancer is the 14th most common cancer worldwide [1] and it is expected to be one of the fastest increasing cancers over the next 20 years [2]. Cancers of the kidney are divided into the transitional cell or renal cell cancers - among the latter, clear cell is the most common subtype. In Trinidad and Tobago, kidney cancer is uncommon, accounting for only 1.2% of new cancer cases between 1995 and 2002. The mortality rate has increased from 1.1 to 1.3% over the same time period [3].

Despite the predicted rise in kidney cancer cases globally, there is a paucity of data on kidney cancer locally. We, therefore, aimed to audit our own experience with kidney cancer in order to document the demographic and pathological profile of renal cancer at San Fernando General Hospital (SFGH) over a five-year period (2015-2019). To the best of our knowledge, this is the first time that a study had documented the pathological profile of renal cancers in Trinidad and Tobago.

The Department of Urology at San Fernando General Hospital is a tertiary referral centre that serves a population of 650,000 but routinely receives patients from throughout Trinidad and Tobago. Service is free to the public, and surgical options for kidney cancers are available including open, and more recently, laparoscopic nephrectomy and nephroureterectomy.

This article was previously presented as a meeting abstract at the Caribbean Urological Association Annual Meeting held between 1st-3rd November 2019, at St. Lucia, West Indies.

## Materials and methods

We retrospectively analyzed the case notes of all patients who had a diagnosis of kidney cancer and who underwent surgical intervention from 2015 to 2019. Patients who were under the age of 18 or those who had missing, or severely limited medical information were excluded from the study. Cases diagnosed clinically without histological confirmation were also excluded.

Clinicopathological data including patient demographics, clinical presentation, and pathological disease characteristics were collected for analysis. The Fuhrman nuclear grade, histological subtype, post-resection tumor margins, nodal status, and the presence of lymphovascular invasion were all included.

All surgeries were performed by trained urology residents and/or urology consultants at our institution. Pathological analyses were conducted by specialist pathologists primarily within our institution using routine hematoxylin and eosin (H and E) stains. However, we did include analyses done by certified pathologists outside the institution if the patient had opted for this route.

Data were collected and tabulated using Microsoft Excel. Descriptive statistics were utilized to summarize the characteristics of our data set. All ethical issues were taken into consideration, including confidentiality and privacy of patient data, and the study was approved by the Campus Research Ethics Committee of the University of the West Indies with approval number CEC1193/07/19.

## Results

During the five-year period, 42 patients had a histological diagnosis of renal cancer, with a greater incidence seen in males. Males comprised 57% and females 43% with a male to female ratio of 1.3:1. Ages of all but three patients were documented and the mean age at detection was 61 years (SD = 14.14, range 18-81).

Clinical presentation and management

Six of the 42 patients lacked information on their clinical presentation to the hospital. Of the remaining 36 patients, 42% had pain either as the sole presenting complaint or in combination with hematuria and/or weight loss, while 30% had hematuria only or hematuria in combination with weight loss. Only weight loss was recorded in 5% of the patients. Other complaints such as lower urinary tract symptoms and fatigue accounted for 5% of clinical presentations. Incidental detection of tumors was recorded in 19% of the cases. 

This study only included patients who had surgical treatment of their disease. The majority, 93%, were treated by radical nephrectomy or nephroureterectomy, and the remaining 7% by partial nephrectomy. The open surgical approach was used in all but three cases where laparoscopic radical nephrectomy was performed instead.

Pathological characteristics of tumors

Of the 42 patients who were found to have renal cancer, 48% had right-sided tumors while 52% were left-sided. The anatomical location of tumors was recorded for 38 of the 42 patients. Of the 38 cases, 39.5% of tumors involved the upper pole of the kidney, while the renal pelvis, middle and lower poles were involved in 18.4%, 15.8%, and 18.4% of cases, respectively. In 7.9% of cases, the tumor was recorded as involving multiple poles.

Renal cell carcinomas (RCC) accounted for 86% of the tumors, with a histological diagnosis of clear cell in 80.5%, papillary in 16.7%, and chromophobe in 2.8%. The remaining six ( 14%) cases were urothelial carcinomas. Of these, there were five high-grade tumors and one low-grade tumor.

Prognostic factors

The surgical margins post-resection were reported in 41 of 42 cases and were found to be negative in 40 out of the 41 cases. There was lymphovascular invasion in only 7% (3 cases). 90.5% of the cases were not assessed for nodal status. Of the four cases that were assessed, only one was positive for lymphatic spread of the tumor (Table 1).

**Table 1:** Status of prognostic factors in renal cancer

Surgical margins (n=41)		Lymphovascular invasion (n=41)		Lymph nodes		
Clear	Involved	Present	Absent	Assessed (9.5%)		Not assessed 38 (90.5%)
				Positive	Negative	
40 (97.6%)	1 (2.4%)	3 (26.8%)	38 (73.2%)	1 (25%)	3 (75%)	

Thirty-four out of the 36 RCC cases were assessed using the Fuhrman nuclear grade. All cases assessed were between grades 1 to 3 with 73% classified as grade 2, 18% as grade 3 and 9% as grade 1. Histologically, all cases of transitional cell carcinoma were urothelial carcinomas of the pelvis with negative tumor margins and no lymphovascular invasion. All of the tumors during this five-year period had a pathological tumor (T)-stage 2 (T2) or less with the average maximum dimension of 4.2 mm; 86% of the tumors had a pathological T-stage 1 (T1) and 14% T2.

## Discussion

Renal cell cancer represents around 3% of all cancers with the highest incidence occurring in western countries. These cancers are more common in men than women with a 1.5:1 male predominance and peak incidence in older individuals [4]. This study had a male to female ratio of 1.3:1 with the mean age of incidence at 61 was in keeping with the international literature.

The most common clinical presentation in our study was flank pain which accounted for 42% of cases, followed by hematuria which occurred in 31% patients. This was compared to work done by Mahasin et al. [5] and Datta et al. [6]. in which flank pain was present in 35% and 73% respectively. In contrast, the most common clinical presentation for renal cancer according to Nardi and colleagues was hematuria which occurred in 43% of cases [7].

Renal cell carcinomas comprise a broad spectrum of histological entities. There are three main types: clear cell (ccRCC), papillary (pRCC), and chromophobe (chRCC), and these were identified in our cohort, with clear cell carcinoma accounting for 80.5% of the RCCs. A study by Datta and colleagues [6] revealed that 86% of renal carcinomas were ccRCC. Similar findings were noted in studies by Mahasin et al. [5] and Nardi and colleagues [7].

Fuhrman nuclear grading is still the most widely accepted grading system for renal cell cancers and was used in this study. The study showed that the most common grade was grade 2, at 74%. Similar results were seen by Mahasin et al. [5] where grade two was also the most common grade. In general, ccRCC has a worse prognosis compared to pRCC and chRCC even after stratification for stage and grade [4]. Indeed, this is hypothesis-generating for a future study at our hospital or even nationally.

All of the tumors assessed had a pathological stage of T2 or less with 86% being T1 and 14% T2. Although similar studies had a wider distribution of T-stages of renal cancer, T-stage 2 or less were the most common. In a study by Nardi and colleagues 32% of patients had pathological T1, 30% T2, 29% T-stage 3 (T3), and 7% T-stage 4 (T4) [7]. A review by Mahasin et al. revealed that 48% of patients had T1 lesions, 22% T2, and 29% T3 [5]. It is likely that advanced-stage disease was under represented in this study given that we only reviewed patients who underwent surgery. Patients with late-stage diseases were less likely to have had nephrectomy and may have been managed clinically. While some may have had a biopsy done, this is not common at our hospital.

Partial nephrectomy (PN) is the reference standard of management for a clinical (c)T1 renal mass [8]. Despite this, 93% of the cases, were treated by radical nephrectomy or nephroureterectomy and the remaining 7% by partial nephrectomy. Additionally, only three cases were operated on laparoscopically, likely due to limited expertise. Making the decision about the best surgical management of a patient is case-dependent. There are many factors that play a role in the decision-making process besides tumor sizes such as life expectancy, comorbidities, anatomy, the difficulty of surgery, and patient wishes; it is likely that this impacted the decision to pursue radical nephrectomy in the majority of these patients, even with an average tumor size of 4.2 cm.

Limitations

This study was retrospective in nature and we were unable to obtain pertinent medical information on all of the patients with a histological diagnosis of renal cell cancer. As a result, persons who had incomplete medical records were excluded and our sample size was negatively affected. This speaks to the need for improved record-keeping within our department. Also, as alluded to above, we did not include patients with more advanced tumors who would not have had a histological diagnosis and this would have biased our study towards lower stage disease. Our study sampled patients from only one, albeit the major, urological referral centre in the country, and individual addresses were not recorded. Our ability to accurately extrapolate our results to the wider population is therefore limited. Nonetheless, this study is a start and will be built upon. A future study on the presence of risk factors, medical treatment, and long-term follow-up to document survival data will be invaluable. Additionally, we do plan to expand this study to other units in order to capture a national sample.

## Conclusions

This study shows that in our hospital renal cancer affects older patients, mostly men, with the common presentation of pain and hematuria. Renal cell carcinomas account for the majority of kidney cancers. The tumors are most commonly clear cell carcinoma, grade 2 with negative margins, no lymphovascular invasion, and an average maximum dimension of 4.2 cm.
